# Correction: Zarate-Calderon et al. Risk of Cerebrovascular Events in Deep Brain Stimulation for Parkinson’s Disease Focused on STN and GPi: Systematic Review and Meta-Analysis. *Brain Sci.* 2025, *15*, 413

**DOI:** 10.3390/brainsci15080838

**Published:** 2025-08-05

**Authors:** Cristofer Zarate-Calderon, Carlos Castillo-Rangel, Iraís Viveros-Martínez, Estefanía Castro-Castro, Luis I. García, Gerardo Marín

**Affiliations:** 1 Institute of Brain Research, Universidad Veracruzana, Xalapa 91190, Mexico; cristoferjzc@gmail.com (C.Z.-C.); irais6013@gmail.com (I.V.-M.);; 2 Department of Neurosurgery, “Hospital Regional 1° de Octubre”, Instituto de Seguridad y Servicios Sociales de los Trabajadores del Estado, Mexico City 07300, Mexico; neuro_cast27@yahoo.com; 3 Faculty of Medicine, Anáhuac Veracruz—Campus Xalapa, Xalapa 91098, Mexico; estefania.67@hotmail.com; 4 Neural Dynamics and Modulation Lab, Cleveland Clinic, Cleveland, OH 44196, USA

## Table Legend

In the original publication, there was a mistake in the legend for Table 1. The original legend required updating due to the following reasons: (1) Previously omitted data was added to Table 1 (specifically concerning Age distinctions, necessitating new abbreviations, WC: with complications, and WOC: without complications). Consequently, the original legend did not include definitions for these new abbreviations. (2) The term “Cochrane risk-of-bias tool” was not consistently presented as its corresponding abbreviation “RoB 2”, which is used elsewhere in the manuscript for consistency. (3) The description for Age used the general term “Average age” without specifying that it could represent either the mean or median age, as reported in the included studies. (4) Other formatting inconsistencies were present (use of ‘:’ instead of ‘=’) [1].

The correct legend appears below. The authors state that the scientific conclusions are unaffected. This correction was approved by the Academic Editor. The original publication has also been updated.

**Table 1.** Characteristics of the selected studies. This table summarizes key information from the studies. Study design: the type of study, where R = retrospective, P = prospective, and RCT = randomized controlled trial. Total number of DBS patients: Number of PD patients undergoing DBS. Structure: The DBS structure where the CVE occurred. Type of CVE: Type of reported CVE, where H = hemorrhage and I = ischemia. Number of CVEs: Total number of CVEs recorded. Quality: Quality assessment score, based on the NOS for all studies, except for Del Bene et al., 2024 [36], was evaluated using the RoB 2. Use of MER: Indicates whether MER was used during DBS surgery. Age: Mean or median age of the overall study population (L = left, R = right, Y = younger, O = older, WO = with complications, WOC = without complications, IQR = interquartile range). Country: The country in which the study was conducted.

## Error in Table

In the original publication, there was a mistake in Table 1 as it was published. (1) While the information in the first few columns was correct, errors were present in the Age and Country columns. Specifically, in the Age column, the data was not ordered correctly corresponding to the respective studies, and two data points were missing. (2) In the Country column, the countries of origin for each study were also not ordered correctly. (3) Furthermore, for consistency with abbreviations used throughout the article, “Gpi” in the Structure column was not presented as “GPi” [1].

The corrected Table 1 appears below.

The authors state that the scientific conclusions are unaffected. This correction was approved by the Academic Editor. The original publication has also been updated.

## Figures and Tables

**Table 1:** Characteristics of the selected studies. This table summarizes key information from the studies. Study design: the type of study, where R = retrospective, P = prospective, and RCT = randomized controlled trial. Total number of DBS patients: Number of PD patients undergoing DBS. Structure: The DBS structure where the CVE occurred. Type of CVE: Type of reported CVE, where H = hemorrhage and I = ischemia. Number of CVEs: Total number of CVEs recorded. Quality: Quality assessment score, based on the NOS for all studies, except for Del Bene et al., 2024 [36], was evaluated using the RoB 2. Use of MER: Indicates whether MER was used during DBS surgery. Age: Mean or median age of the overall study population (L = left, R = right, Y = younger, O = older, WO = with complications, WOC = without complications, IQR = interquartile range). Country: The country in which the study was conducted.

Author and Year	Study Design	Total Number of DBS Patients	Structure	Type of CVE	Number of CVE	Quality	Use of MER	Age	Country
Seijo et al., 2014 [19]	R	233	STN	H	10	7	YES	61.09 (7.8)	Spain
Tonge et al., 2015 [10]	R	137	-	H	3	7	YES	57.0 (13.6)	Netherlands and Turkey
Downes et al., 2016 [20]	R	112	GPi	I	4	7	YES	60.3 (14.3)	United States
Petraglia et al., 2016 [21]	R	713	-	H	20	9	-	61.1 (9.9)	United States
Cui et al., 2016 [22]	P	110	STN	H	2	6	YES	58 (IQR: 39–77)	China
Park et al., 2017 [9]	R	136	STN	H	9	7	YES	55.14 (13.8)	South Korea
Wang et al., 2017 [23]	R	318	STN	H	10	7	NO	55.7 (14.8)	China
Ryu et al., 2017 [24]	R	42	STN and GPi	H and I	2	8	-	STN: 56.9 (7.7); GPi: 57.9 (8.4)	South Korea
Kim et al., 2018 [25]	R	55	STN	H	1	7	YES	Y: 56.7 (5.7); O: 68.5 (2.9)	South Korea
Koivu et al., 2018 [26]	R	87	STN	H	1	6	NO	61 (IQR: 54–65)	Finland
Sharma et al., 2019 [27]	P	30	STN	H	1	7	YES	77.5 (2.1)	United States
Sobstyl et al., 2019 [11]	R	186	-	H	7	7	NO	-	Poland
Mitchell et al., 2020 [28]	R	104	STN	H	4	9	YES	Y: 60.8 (7.1); O: 77.6 (2.8)	United States
Yang et al., 2020 [29]	R	352	STN and GPi	H	11	7	-	62.22 (6.08)	China
Cordeiro et al., 2020 [30]	R	152	-	H	2	7	YES	64.7 (10.4)	United States
Jung et al., 2022 [31]	R	315	-	H	9	6	YES	57.5 (11.9)	South Korea
Jiang et al., 2022 [32]	R	21	STN and GPi	H	1	7	YES	75 (IQR: 75–85)	China
Shin et al., 2022 [33]	R	250	-	H	11	9	YES	-	South Korea
Servello et al., 2023 [34]	R	517	STN and GPi	H	13	7	YES	61 (IQR: 35–77)	Italy
Mainardi et al., 2024 [35]	R	48	STN and GPi	I	1	9	-	STN: 58 (IQR 14); GPi: 61 (IQR 11.5)	Italy
Del Bene et al., 2024 [36]	RCT	31	STN	H	2	Low Risk	YES	L-STN: 56.7 (8.6); R-STN: 58.9 (6.3)	United States
Eiamcharoenwit et al., 2024 [37]	R	46	STN	H	3	7	YES	WC: 58 (IQR: 53–66); WOC: 59 (IQR: 55–65)	Thailand
Holewijn et al., 2024 [38]	R	800	STN	H and I	23	7	-	61.1 (8.4)	Netherlands
