# Supplementary material for: Risk of Cerebrovascular Events in Deep Brain Stimulation for Parkinson’s Disease Focused on STN and GPi: Systematic Review and Meta-Analysis
Source: Brain Sci. 2025 Apr 18;15(4):413. doi: 10.3390/brainsci15040413 (PMC12026004; doi:10.3390/brainsci15040413)
Supplement: Supplementary file 1 [file brainsci-15-00413-s001.zip › supplementary material 1.pdf]

Supplementary Material File S1

Topic	Search engine	Booleans	Initial search	2014-2024	First 10 pages of search engine	Title and content	No duplicate articles	Selected articles
Parkinson's disease and DBS - CVD risk	Pubmed	("Deep Brain Stimulation") AND ("hemorrhage" OR "stroke" OR "ischemia" OR "thrombosis") AND ("Parkinson disease")	199	123	100	14	38	23
	Cochrane library	("Deep Brain Stimulation") AND ("hemorrhage" OR "stroke" OR "ischemia" OR "thrombosis") AND ("Parkinson disease")	33	23	23	0		
	Google Scholar	("Deep Brain Stimulation") AND ("hemorrhage" OR "stroke" OR "ischemia" OR "thrombosis") AND ("Parkinson disease")	24500	16400	100	13		
	SciELO	"Deep Brain Stimulation" AND ("hemorrhage" OR "stroke" OR "ischemia" OR "thrombosis") AND "Parkinson disease"	0	0	0	0		
	Scopus	("Deep Brain Stimulation") AND ("hemorrhage" OR "stroke" OR "ischemia" OR "thrombosis") AND ("Parkinson disease")	8464	6255	100	1		
Parkinson's disease and DBS - General vascular disorders	Pubmed	("Parkinson's disease") AND "Deep Brain Stimulation" AND ("Intracerebral hemorrhage" OR "Subarachnoid hemorrhage" OR "Intraventricular hemorrhage" OR "Cerebral ischemia" OR "Cerebral venous thrombosis" OR "Stroke")	161	122	100	6		
	Cochrane library	("Parkinson's disease") AND "Deep Brain Stimulation" AND ("Intracerebral hemorrhage" OR "Subarachnoid	12	9	9	0		

		hemorrhage" OR "Intraventricular hemorrhage" OR "Cerebral ischemia" OR "Cerebral venous thrombosis" OR "Stroke")						
	Google Scholar	("Parkinson's disease") AND "Deep Brain Stimulation" AND ("Intracerebral hemorrhage" OR "Subarachnoid hemorrhage" OR "Intraventricular hemorrhage" OR "Cerebral ischemia" OR "Cerebral venous thrombosis" OR "Stroke")	474	7080	100	2		
	SciELO	Parkinson's disease AND "Deep Brain Stimulation" AND ("Intracerebral hemorrhage" OR "Subarachnoid hemorrhage" OR "Intraventricular hemorrhage" OR "Cerebral ischemia" OR "Cerebral venous thrombosis" OR "Stroke")	0	0	0	0		
	Scopus	("Parkinson's disease") AND "Deep Brain Stimulation" AND ("Intracerebral hemorrhage" OR "Subarachnoid hemorrhage" OR "Intraventricular hemorrhage" OR "Cerebral ischemia" OR "Cerebral venous thrombosis" OR "Stroke")	7067	5366	100	1		
DBS in STN/GP - CVD risk	Pubmed	("Deep Brain Stimulation") AND ("hemorrhage" OR "stroke" OR "ischemia" OR "thrombosis") AND ("subthalamic nucleus" OR "globus pallidus")	203	120	100	8		
	Cochrane library	("Deep Brain Stimulation") AND ("hemorrhage" OR "stroke" OR "ischemia" OR "thrombosis") AND ("subthalamic nucleus" OR "globus pallidus")	26	18	18	1		
	Google Scholar	("Deep Brain Stimulation") AND ("hemorrhage" OR "stroke" OR "ischemia"	17400	12300	100	5		

		OR "thrombosis") AND ("subthalamic nucleus" OR "globus pallidus")						
	SciELO	Deep Brain Stimulation AND ("hemorrhage" OR "stroke" OR "ischemia" OR "thrombosis") AND ("subthalamic nucleus" OR "globus pallidus")	0	0	0	0		
	Scopus	("Deep Brain Stimulation") AND ("hemorrhage" OR "stroke" OR "ischemia" OR "thrombosis") AND ("subthalamic nucleus" OR "globus pallidus")	4838	3245	100	2		
DBS - CVD and Adverse effects	Pubmed	("Intracerebral hemorrhage" OR "Subarachnoid hemorrhage" OR "Intraventricular hemorrhage" OR "Cerebral ischemia" OR "Cerebral venous thrombosis" OR "Stroke" OR "Adverse effects") AND ("Deep Brain Stimulation")	2935	1618	100	3		
	Cochrane library	("Intracerebral hemorrhage" OR "Subarachnoid hemorrhage" OR "Intraventricular hemorrhage" OR "Cerebral ischemia" OR "Cerebral venous thrombosis" OR "Stroke" OR "Adverse effects") AND ("Deep Brain Stimulation")	1	1	1	0		
	Google Scholar	("Intracerebral hemorrhage" OR "Subarachnoid hemorrhage" OR "Intraventricular hemorrhage" OR "Cerebral ischemia" OR "Cerebral venous thrombosis" OR "Stroke" OR "Adverse effects") AND ("Deep Brain Stimulation")	51400	19400	100	4		
	SciELO	("Intracerebral hemorrhage" OR "Subarachnoid hemorrhage" OR "Intraventricular hemorrhage" OR "Cerebral ischemia" OR "Cerebral venous thrombosis" OR "Stroke" OR "Adverse effects") AND "Deep Brain Stimulation"	4	2	2	0		
	Scopus	("Intracerebral hemorrhage" OR "Subarachnoid hemorrhage" OR	13555	10301	100	1		

		"Intraventricular hemorrhage" OR "Cerebral ischemia" OR "Cerebral venous thrombosis" OR "Stroke" OR "Adverse effects") AND ("Deep Brain Stimulation")						
DBS - Vascular complications	Pubmed	("Complications" OR "postoperative complications") AND ("Deep Brain Stimulation") AND ("vascular injury" OR "thrombosis" OR "vascular disorders" OR "hemorrhage" OR "ischemia" OR "Stroke")	312	189	100	13		
	Cochrane library	("Complications" OR "postoperative complications") AND ("Deep Brain Stimulation") AND ("vascular injury" OR "thrombosis" OR "vascular disorders" OR "hemorrhage" OR "ischemia" OR "Stroke")	1	1	1	0		
	Google Scholar	("Complications" OR "postoperative complications") AND ("Deep Brain Stimulation") AND ("vascular injury" OR "thrombosis" OR "vascular disorders" OR "hemorrhage" OR "ischemia" OR "Stroke")	21300	14200	100	4		
	SciELO	("Complications" OR "postoperative complications") AND "Deep Brain Stimulation" AND ("vascular injury" OR "thrombosis" OR "vascular disorders" OR "hemorrhage" OR "ischemia" OR "Stroke")	0	0	0	0		
	Scopus	("Complications" OR "postoperative complications") AND ("Deep Brain Stimulation") AND ("vascular injury" OR "thrombosis" OR "vascular disorders" OR "hemorrhage" OR "ischemia" OR "Stroke")	3965	2971	100	1		
Parkinson's disease in STN/GP - Vascular complications	Pubmed	("Parkinson's disease") AND ("STN" OR "GP") AND ("adverse effects" OR "vascular complications") AND ("Deep Brain Stimulation")	474	237	100	4		
	Cochrane library	("Parkinson's disease") AND ("STN" OR "GP") AND ("adverse effects" OR "vascular	59	39	39	1		

		complications") AND ("Deep Brain Stimulation")						
	Google Scholar	("Parkinson's disease") AND ("STN" OR "GP") AND ("adverse effects" OR "vascular complications") AND ("Deep Brain Stimulation")	98	50	50	0		
	SciELO	Parkinson's disease AND ("STN" OR "GP") AND ("adverse effects" OR "vascular complications") AND "Deep Brain Stimulation"	0	0	0	0		
	Scopus	("Parkinson's disease") AND ("STN" OR "GP") AND ("adverse effects" OR "vascular complications") AND ("Deep Brain Stimulation")	707	470	100	1		

Table S1. Literature search results and search parameters. Summary of the systematic search strategy and results across different databases (PubMed, Cochrane Library, Google Scholar, SciELO, and Scopus). Search parameters include Boolean terms and specific keywords related to Deep Brain Stimulation (DBS), Parkinson's disease (PD), and associated vascular risks and complications. The table details the number of initial search results, duplicates removed, and selected articles.

ID	Articles	Selected
1	A Detailed Analysis of Intracerebral Hemorrhages in DBS Surgeries	1
2	Acute Ischemic Stroke During Deep Brain Stimulation Surgery of Globus Pallidus Internus: Report of 5 Cases	1
3	Analysis of Delayed Intracerebral Hemorrhage Associated with Deep Brain Stimulation Surgery	1
4	Awake Versus Asleep Deep Brain Stimulation Targeting the Caudal Zona Incerta for Essential Tremor	-
5	Bilateral Subthalamic Deep Brain Stimulation is an Effective and Safe Treatment Option for the Older Patients with Parkinson's Disease	1
6	Bilateral Subthalamic Nucleus Deep Brain Stimulation in Elderly Patients with Parkinson Disease: A Case-Control Study	1
7	Clinical Analysis and Treatment of Symptomatic Intracranial Hemorrhage After Deep Brain Stimulation Surgery	1
8	Comparison of Bilateral Versus Staged Unilateral Deep Brain Stimulation in Parkinson's Disease in Patients Under 70 Years of Age	1
9	Comparison of Pallidal and Subthalamic Deep Brain Stimulation in Parkinson's Disease: Therapeutic and Adverse Effects	1
10	Complications After Deep Brain Stimulation: A 21-Year Experience in 426 Patients	1
11	Complications of Deep Brain Stimulation in Parkinson's Disease: A Single-Center Experience of 517 Consecutive Cases	1
12	Deep Brain Stimulation of Globus Pallidus Internus and Subthalamic Nucleus in Parkinson's Disease: A Multicenter, Retrospective Study of Efficacy and Safety	1
13	Deep Brain Stimulation of the Subthalamic Nucleus in Parkinson's Disease Patients Over 75 Years of Age	1
14	Differential Cognitive Effects of Unilateral Subthalamic Nucleus Deep Brain Stimulation for Parkinson's Disease	1
15	Effect of Advancing Age on Outcomes of Deep Brain Stimulation for Parkinson Disease	-
16	Hemorrhage Detection and Incidence During Magnetic Resonance-Guided Deep Brain Stimulator Implantations	-
17	Hemorrhagic Complications Seen on Immediate Intraprocedural Stereotactic Computed Tomography Imaging During Deep Brain Stimulation Implantation	1
18	Incidence of Complications Associated with Deep Brain Stimulation Surgery in Patients with Parkinson's Disease: An 8-Year Retrospective Study	1
19	Intracerebral Hemorrhage and Venous Infarction After Deep Brain Stimulation Lead Placement	-
20	Intracranial Hemorrhage Risk Factors of Deep Brain Stimulation for Parkinson's Disease: A 2-Year Follow-Up Study	1
21	Intra-operative Micro-electrode Recording in Functional Neurosurgery: Past, Present, Future	-

22	Intraoperative MRI for Optimizing Electrode Placement for Deep Brain Stimulation of the Subthalamic Nucleus in Parkinson Disease	1
23	Intravenous Thrombolysis and Mechanical Recanalization for Acute Ischemic Stroke in Deep Brain Stimulation Patients: A Case Series	-
24	Long-Term Evaluation of Changes in Operative Technique and Hardware-Related Complications With Deep Brain Stimulation	-
25	Minimising the Rate of Vascular Complications in Deep Brain Stimulation Surgery for the Management of Parkinson's Disease: A Single-Centre 600-Patient Case Series	-
26	Motor Outcome and Electrode Location in Deep Brain Stimulation in Parkinson's Disease	1
27	Nomogram Model for Predicting Risk of Postoperative Delirium in Parkinson's Patients Over 50 Years Old After Deep Brain Stimulation Surgery	-
28	Perioperative Complications of Deep Brain Stimulation Among Patients with Advanced Age: A Single-Institution Retrospective Analysis	-
29	Quality of Life and Motor Outcomes in Patients with Parkinson's Disease 12 Months After Deep Brain Stimulation in China	-
30	Risks of Common Complications in Deep Brain Stimulation Surgery: Management and Avoidance	-
31	Safety of Non-Contrast Imaging Guided DBS Electrode Placement in Parkinson's Disease	1
32	Short- and Long-Term Efficacy and Safety of Deep-Brain Stimulation in Parkinson's Disease Patients aged 75 Years and Older	1
33	Subthalamic Nucleus Deep Brain Stimulation Treats Parkinson's Disease Patients with Cardiovascular Disease Comorbidity: A Retrospective Study of a Single Center Experience	-
34	Surgical Adverse Events of Deep Brain Stimulation in the Subthalamic Nucleus of Patients with Parkinson's Disease: The Learning Curve and the Pitfalls	1
35	Surgical and Hardware Complications of Deep Brain Stimulation—A Single Surgeon Experience of 519 Cases Over 20 Years	-
36	Surgical Complications in Subthalamic Nucleus Deep Brain Stimulation for Parkinson's Disease: Experience in 800 Patients	1
37	The Impact of Microelectrode Recording on Lead Location in Deep Brain Stimulation for the Treatment of Movement Disorders	-
38	The risk factors of intracerebral hemorrhage in deep brain stimulation: does target matter?	1

Table S2. Selected articles for systematic review. List of articles selected after the systematic review process, focusing on studies related to Deep Brain Stimulation (DBS) in Parkinson's disease and its associated vascular complications. Includes article titles to provide an overview of the scope and relevance of the studies. The number 1 in the Selected column indicates that the article was selected.

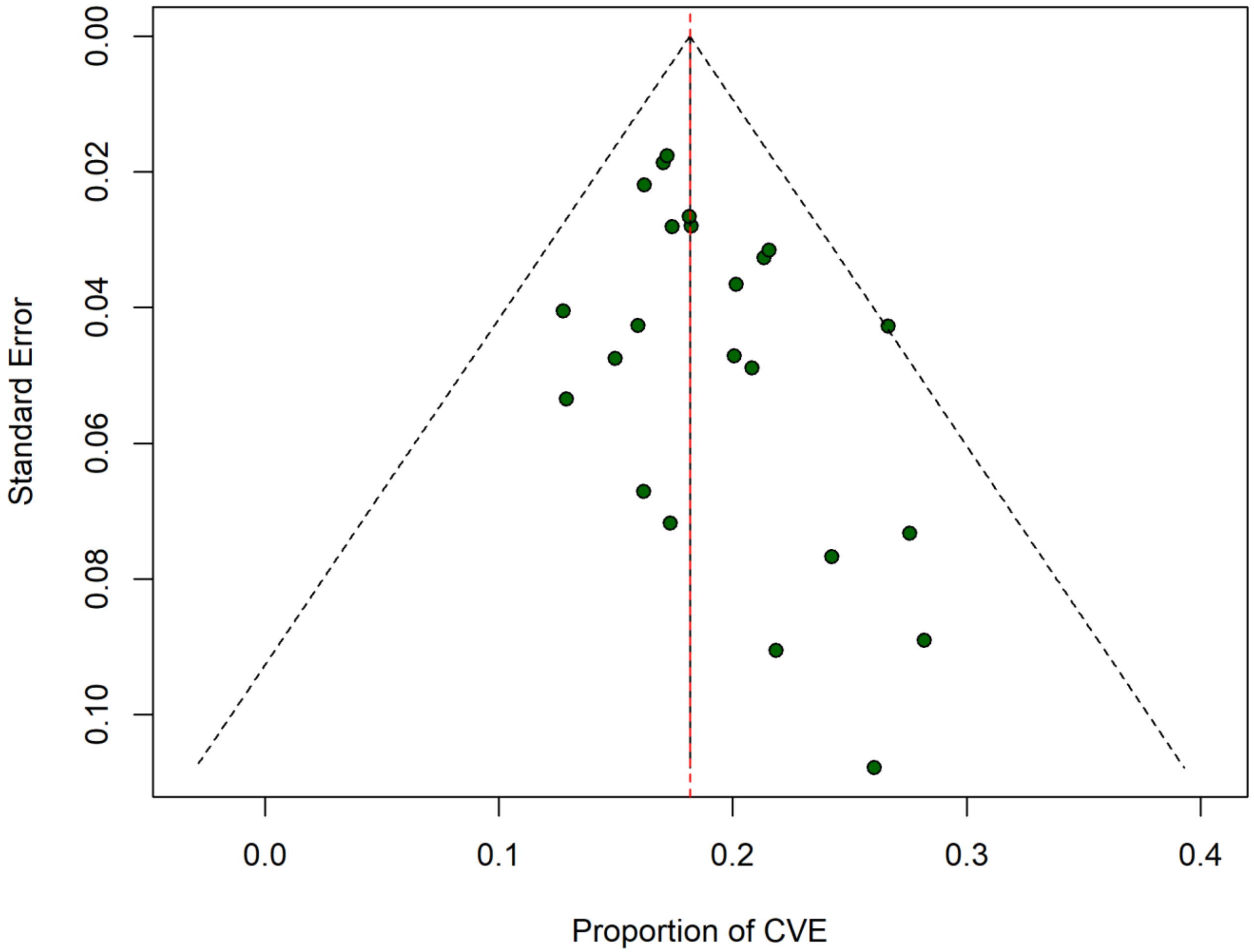

Figure S1. General assessment of publication bias. Funnel plot assessing publication bias in the meta-analysis of CVE in PD patients undergoing DBS.

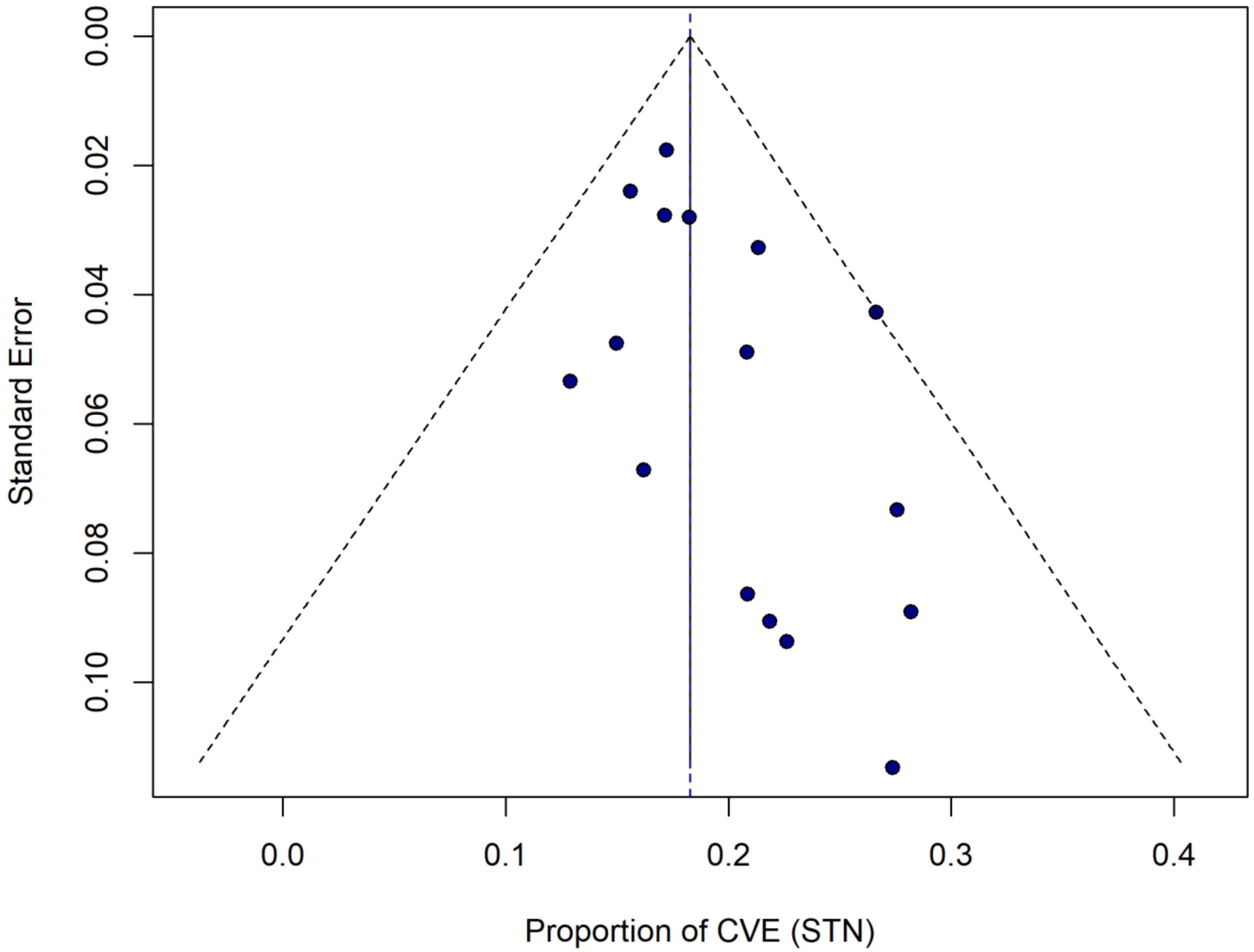

Figure S2. Publication bias in STN analysis. Funnel plot assessing publication bias in the meta-analysis of CVE probability for the DBS-STN.

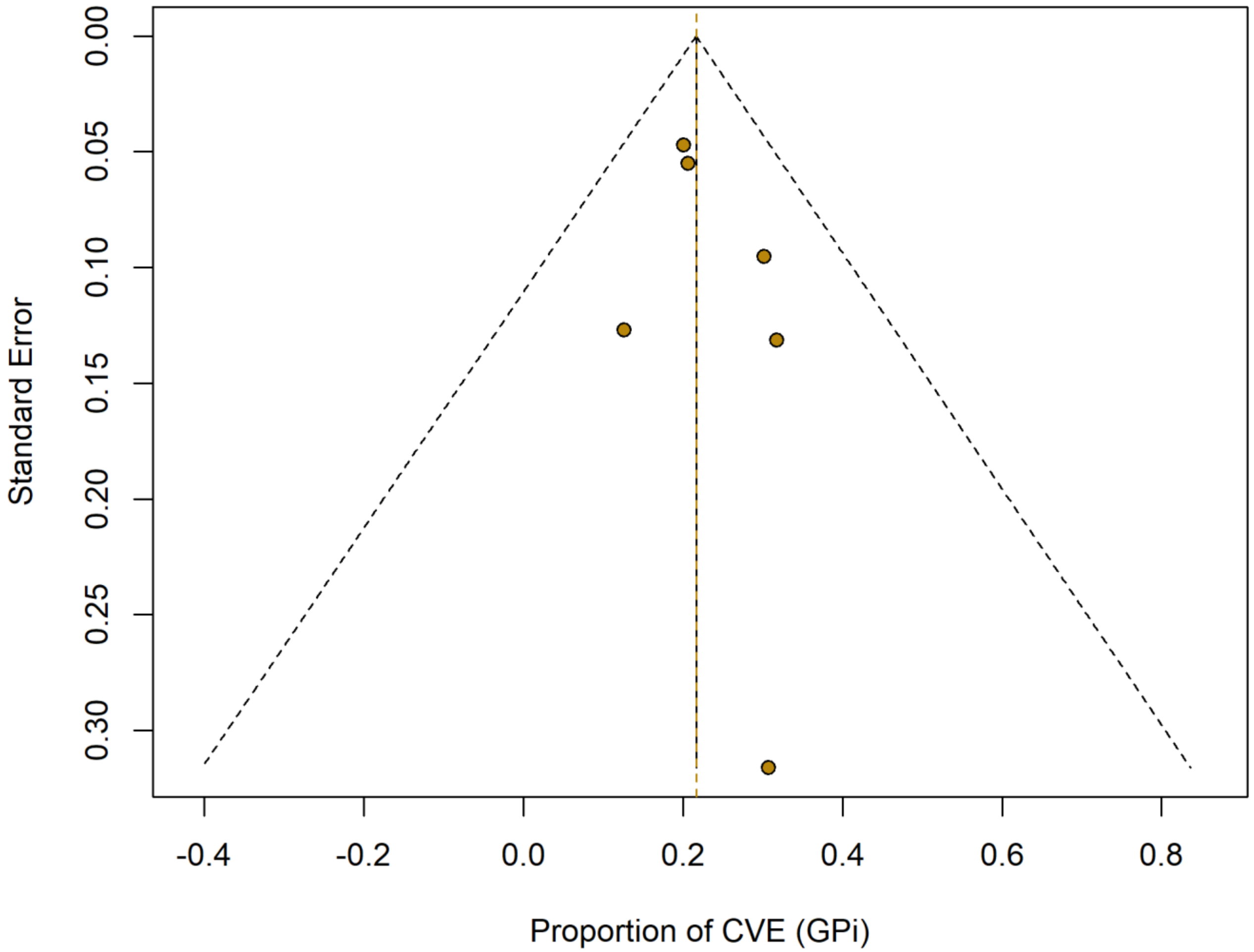

Figure S3. Publication bias in GPi analysis. Funnel plot assessing publication bias in the meta-analysis of CVE probability for the DBS-GPi.

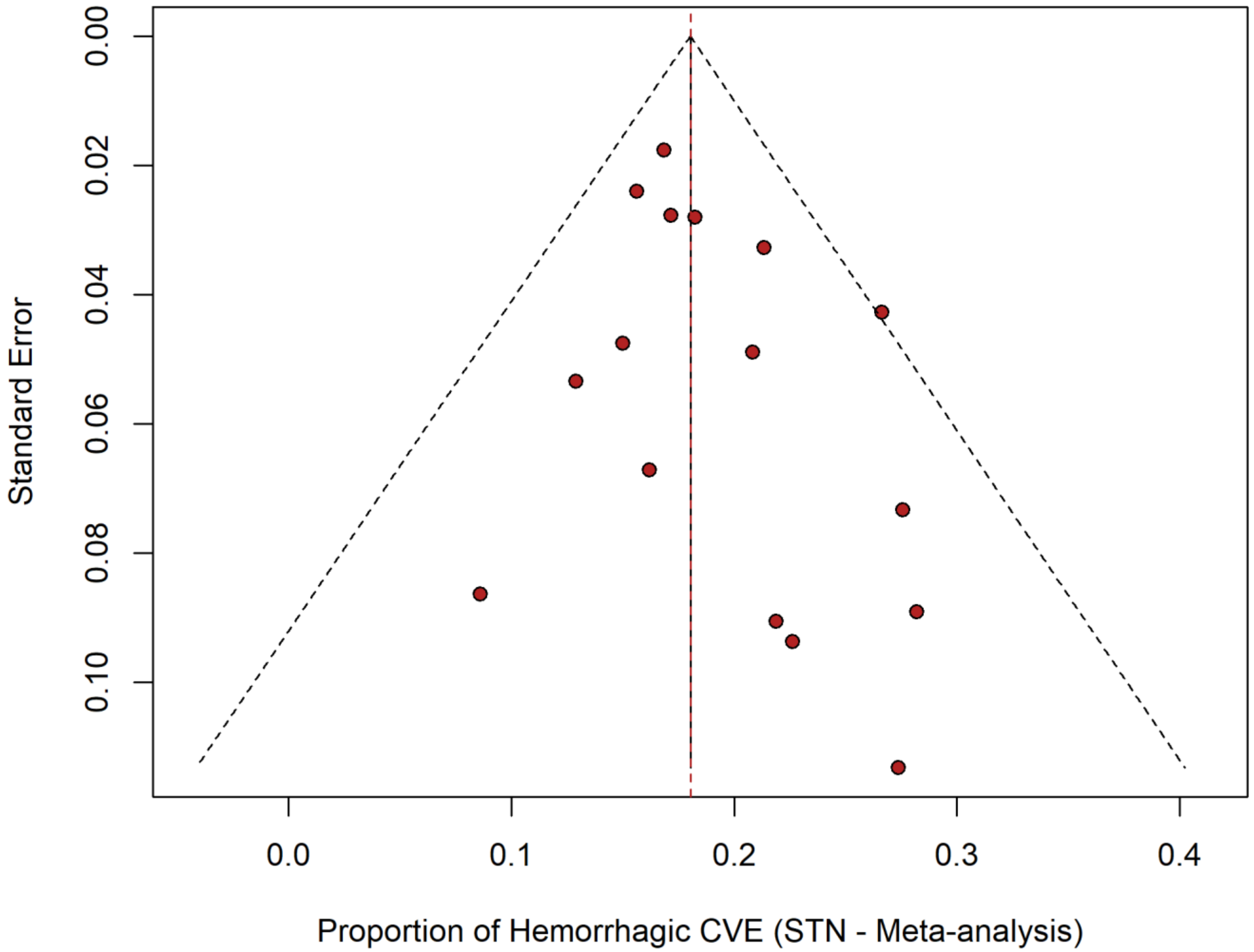

Figure S4. Publication bias in STN hemorrhagic analysis. Funnel plot assessing publication bias in the meta-analysis of hemorrhage probability for DBS-STN.

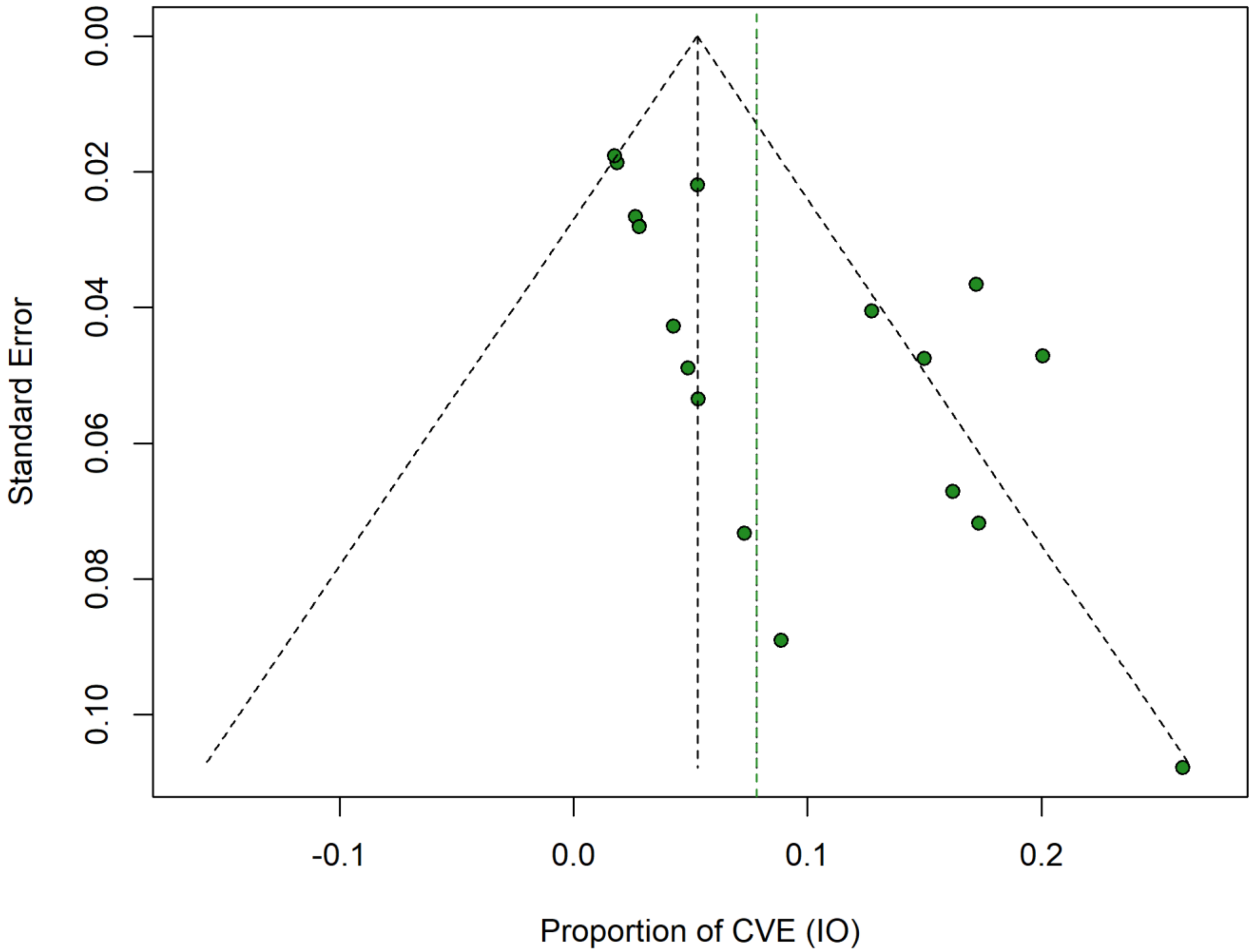

Figure S5. Publication bias in IO CVEs. Funnel plot assessing publication bias in the meta-analysis of probability for intraoperative CVEs.

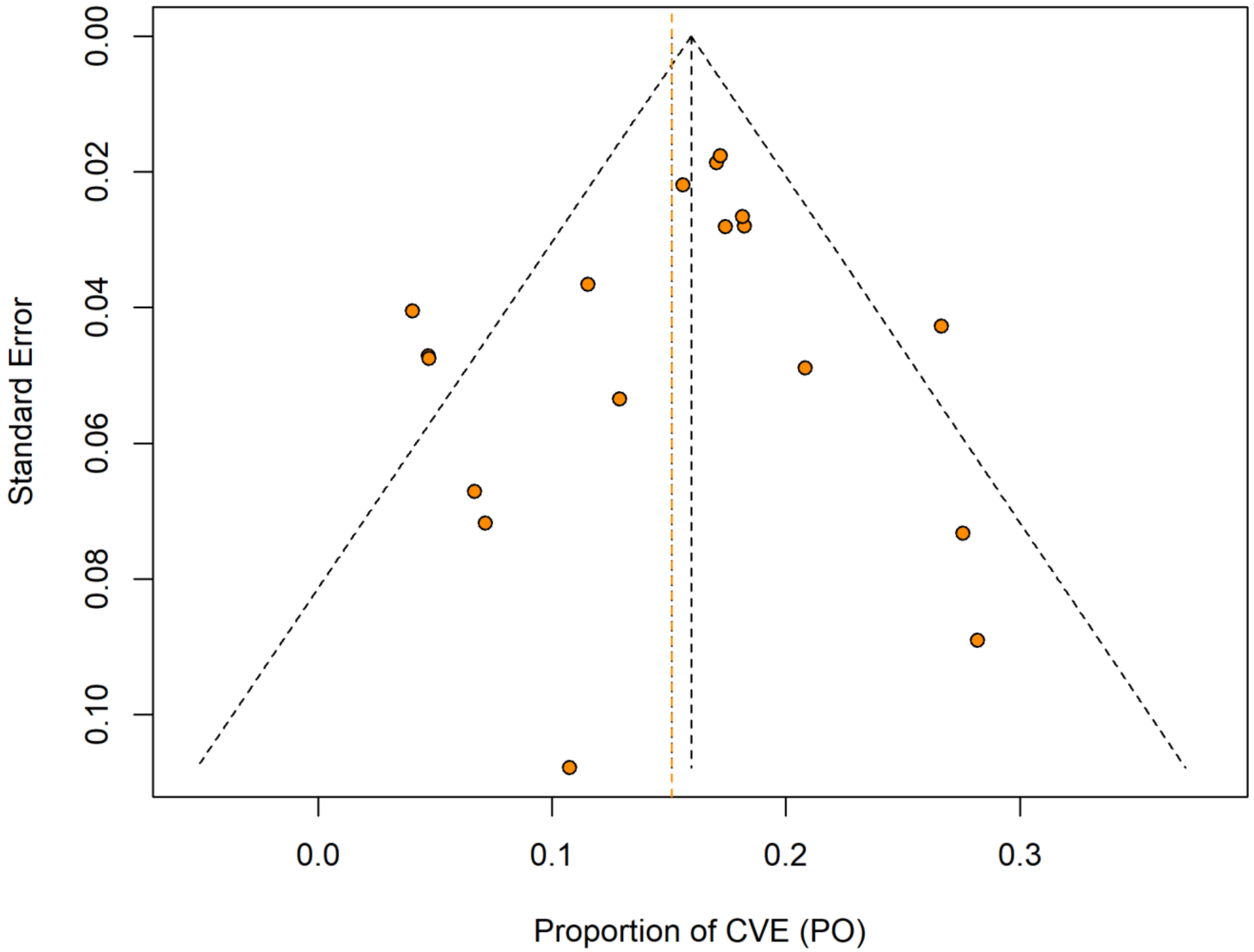

Figure S6. Publication bias in PO CVEs. Funnel plot assessing publication bias in the meta-analysis of probability for postoperative CVEs.

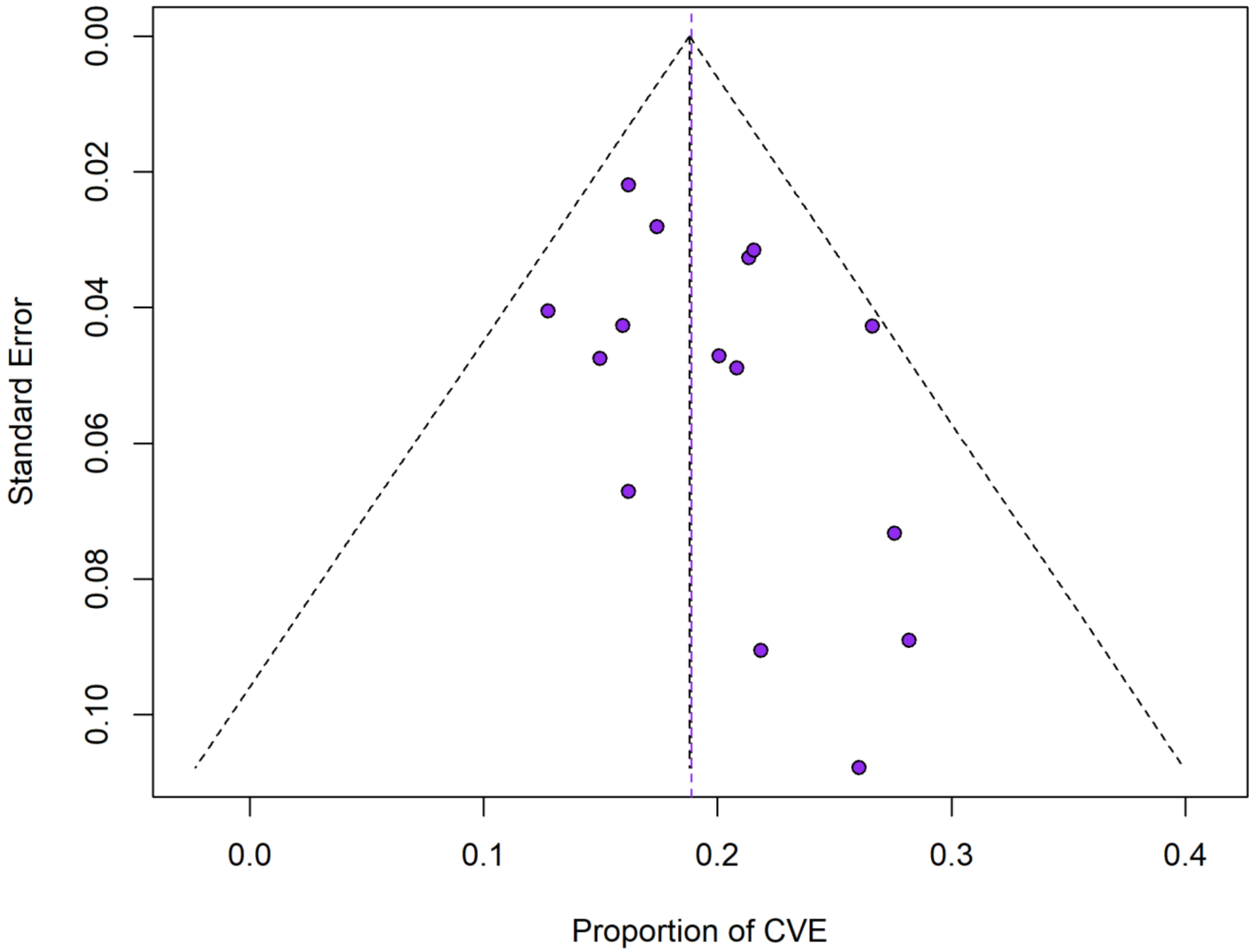

Figure S7. Publication bias in MER CVEs. Funnel plot assessing publication bias in the meta-analysis of probability for CVEs using the MER technique.

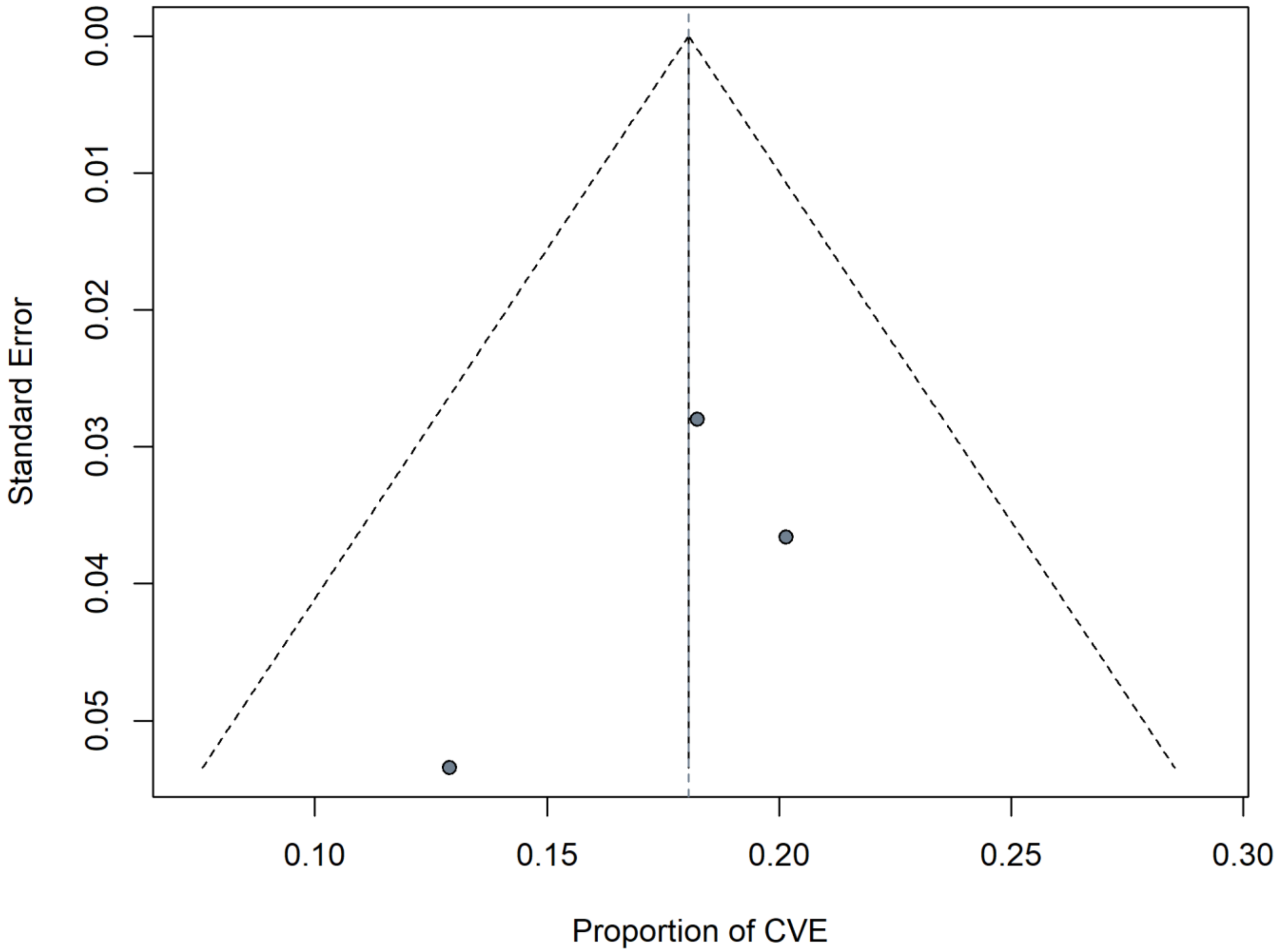

Figure S8. Publication bias in NON-MER CVEs. Funnel plot assessing publication bias in the meta-analysis of probability for CVEs without using MER technique.
