# Supplementary material for: Risk of Cerebrovascular Events in Deep Brain Stimulation for Parkinson’s Disease Focused on STN and GPi: Systematic Review and Meta-Analysis
Source: Brain Sci. 2025 Apr 18;15(4):413. doi: 10.3390/brainsci15040413 (PMC12026004; doi:10.3390/brainsci15040413)
Supplement: Supplementary file 1 [file brainsci-15-00413-s001.zip › supplementary material 2.pdf]

**Supplementary Material File S2**

| AUTHOR | YEAR | N_TOTAL | HEM | ISCH | TOTAL_CVE | STN-DBS | GPi-DBS | STN_H | STN_I | GPi_H | GPi_I | IO | PO | MER | NON-MER | MER_TOTAL | NON-MER_TOTAL | AGE | NACIONALITY |
|---|---|---|---|---|---|---|---|---|---|---|---|---|---|---|---|---|---|---|---|
| Seijo et al. | 2014 | 233 | 10 | 0 | 10 | 233 | - | 10 | 0 | - | - | - | - | 10 | 0 | 233 | 0 | 61.09 (7.8) | Spain |
| Tonge et al. | 2015 | 137 | 3 | 0 | 3 | - | - | - | - | - | - | - | - | 3 | 0 | 137 | 0 | 57.0 (13.6) | Netherlands and Turkey |
| Downes et al. | 2016 | 112 | 0 | 4 | 4 | - | 112 | - | - | 0 | 4 | 4 | 0 | 4 | 0 | 112 | 0 | 60.3 (14.3) | United States |
| Petraglia et al. | 2016 | 713 | 20 | 0 | 20 | - | - | - | - | - | - | 0 | 20 | - | - | - | - | 61.1 (9.9) | United States |
| Cui et al. | 2016 | 110 | 2 | 0 | 2 | 110 | - | 2 | 0 | - | - | 2 | 0 | 2 | 0 | 110 | 0 | 58 (IQR: 39-77) | China |
| Park et al. | 2017 | 136 | 9 | 0 | 9 | 136 | - | 9 | 0 | - | - | 0 | 9 | 9 | 0 | 136 | 0 | 55.14 (13.8) | South Korea |
| Wang et al. | 2017 | 318 | 10 | 0 | 10 | 318 | - | 10 | 0 | - | - | 0 | 10 | 0 | 10 | 0 | 318 | 55.7 (14.8) | China |
| Ryu et al. | 2017 | 42 | 1 | 1 | 2 | 28 | 14 | 1 | 0 | 0 | 1 | - | - | - | - | - | - | STN: 56.9 (7.7);<br>GPi: 57.9 (8.4) | South Korea |
| Kim et al. | 2018 | 55 | 1 | 0 | 1 | 55 | - | 1 | 0 | - | - | 1 | 0 | 1 | 0 | 55 | 0 | Y: 56.7 (5.7);<br>O: 68.5 (2.9) | South Korea |
| Koivu et al. | 2018 | 87 | 1 | 0 | 1 | 87 | - | 1 | 0 | - | - | 0 | 1 | 0 | 1 | 0 | 87 | 61 (IQR: 54 - 65) | Finland |
| Sharma et al. | 2019 | 30 | 1 | 0 | 1 | 30 | - | 1 | 0 | - | - | - | - | 1 | 0 | 30 | 0 | 77.5 (2.1) | United States |
| Sobstyl et al. | 2019 | 186 | 7 | 0 | 7 | - | - | - | - | - | - | 5 | 2 | 0 | 7 | 0 | 186 | - | Poland |
| Mitchell et al. | 2020 | 104 | 4 | 0 | 4 | 104 | - | 4 | 0 | - | - | 0 | 4 | 4 | 0 | 104 | 0 | Y: 60.8 (7.1);<br>O: 77.6 (2.8) | United States |
| Yang et al. | 2020 | 352 | 11 | 0 | 11 | 325 | 27 | 9 | 0 | 2 | 0 | 0 | 11 | - | - | - | - | 62.22 (6.08) | China |
| Cordeiro et al. | 2020 | 152 | 2 | 0 | 2 | - | - | - | - | - | - | 2 | 0 | 2 | 0 | 152 | 0 | 64.7 (10.4) | United States |
| Jung et al. | 2022 | 315 | 9 | 0 | 9 | - | - | - | - | - | - | 0 | 9 | 9 | 0 | 315 | 0 | 57.5 (11.9) | South Korea |
| Jiang et al. | 2022 | 21 | 1 | 0 | 1 | 19 | 2 | 1 | 0 | 0 | 0 | 1 | 0 | 1 | 0 | 21 | 0 | 75 (IQR: 75-85) | China |
| Shin et al. | 2022 | 250 | 11 | 0 | 11 | - | - | 9 | - | 2 | - | - | - | 11 | 0 | 250 | 0 | - | South Korea |
| Servello et al. | 2023 | 517 | 13 | 0 | 13 | 433 | 82 | 10 | 0 | 3 | 0 | 1 | 12 | 13 | 0 | 517 | 0 | 61 (IRQ: 35-77) | Italy |
| Mainardi et al. | 2024 | 48 | 0 | 1 | 1 | 33 | 15 | 0 | 1 | 0 | 0 | 1 | 0 | - | - | - | - | STN: 58 (IQR 14);<br>GPi: 61 (IQR 11.5) | Italy |
| Del Bene et al. | 2024 | 31 | 2 | 0 | 2 | 31 | - | 2 | 0 | - | - | 0 | 2 | 2 | 0 | 31 | 0 | L: STN: 56.7 (8.6);<br>R: STN: 58.9 (6.3) | United States |
| Eiamcharoenwit et al. | 2024 | 46 | 3 | 0 | 3 | 46 | - | 3 | 0 | - | - | 0 | 3 | 3 | 0 | 46 | 0 | W: 58 (IRQ: 53-66);<br>WO: 59 (IRQ: 55-65) | Thailand |
| Holewijn et al. | 2024 | 800 | 22 | 1 | 23 | 800 | - | 22 | 1 | - | - | 0 | 23 | - | - | - | - | 61.1 (8.4) | Netherlands |

**Table S3.** Database extracted from selected articles. This table presents a detailed dataset extracted from the selected articles in the systematic review. The columns include: AUTHORS, referring to the authors of each study; YEAR, the year of publication; N_TOTAL, the total number of PD patients undergoing DBS; HEM, the number of reported hemorrhages; ISCH, the number of reported ischemic events; TOTAL_CVE, the total number of cerebrovascular events reported; STN_H and GPi_H, the number of hemorrhages reported in the STN or GPi, respectively; STN_I and GPi_I, the number of ischemic events reported in the STN or GPi, respectively; IO, the number of intraoperative CVEs; PO, the number of postoperative CVEs; MER, the number of CVEs in which MER was used; NON-MER, the number of CVEs in which MER was not used; MER_TOTAL, the total number of PD patients undergoing DBS in whom MER was used; NON-MER_TOTAL, the total number of PD patients undergoing DBS in whom MER was not used; AGE, the mean or median age reported for the PD-DBS population (L: left, R: right, Y: younger, O: older, W: with complications, WO: without complications , IQR: interquartile range); and NACIONALITY, the country of origin from which the data were extracted.
